# (OTA)_3_FeMo_6_-DES: a liquid catalyst integrating catalytic and extractive functions for room-temperature oxidative desulfurization

**DOI:** 10.1039/d6ra02289h

**Published:** 2026-11-11

**Authors:** Yunying Bai, Yang Hua, Yuan Shen, Xin Meng, Yufan Yang, Hong Zhang

**Affiliations:** a Key Laboratory of Polyoxometalate and Reticular Material Chemistry of Ministry of Education, Department of Chemistry, Northeast Normal University Changchun Jilin 130024 PR China zhangh@nenu.edu.cn

## Abstract

To address the intrinsic limitations of up-to-date oxidative desulfurization, this study adopted a synergistic polyoxometalate (POM) – deep eutectic solvent (DES) catalytic strategy to prepare a liquid catalyst (POM-DES) with dual functions of catalysis and extraction through simple stirring. Experimental results demonstrate that the catalyst—composed of an Anderson-type polyoxometalate (OTA)_3_FeMo_6_ and a *p*-toluene sulfonic acid-based ternary DES—achieves complete removal of DBT under mild conditions (30 °C, O/S = 2.8, DES dosage = 0.5 mL, POM loading = 0.02 g mL^−1^) within 30 minutes. Moreover, the catalyst exhibits excellent recyclability, maintaining high activity over five consecutive cycles without significant deactivation. This work not only presents an efficient and cost-effective strategy for deep desulfurization under mild conditions but also offers new insights into the design and optimization of multicomponent cooperative catalytic systems.

## Abstract 2

To overcome current oxidative desulfurization limitations, a synergistic polyoxometalate – deep eutectic solvent (POM–DES) strategy was used to prepare a liquid catalyst (POM-DES) with dual catalytic and extraction functions *via* simple stirring.

## Graphical abstract

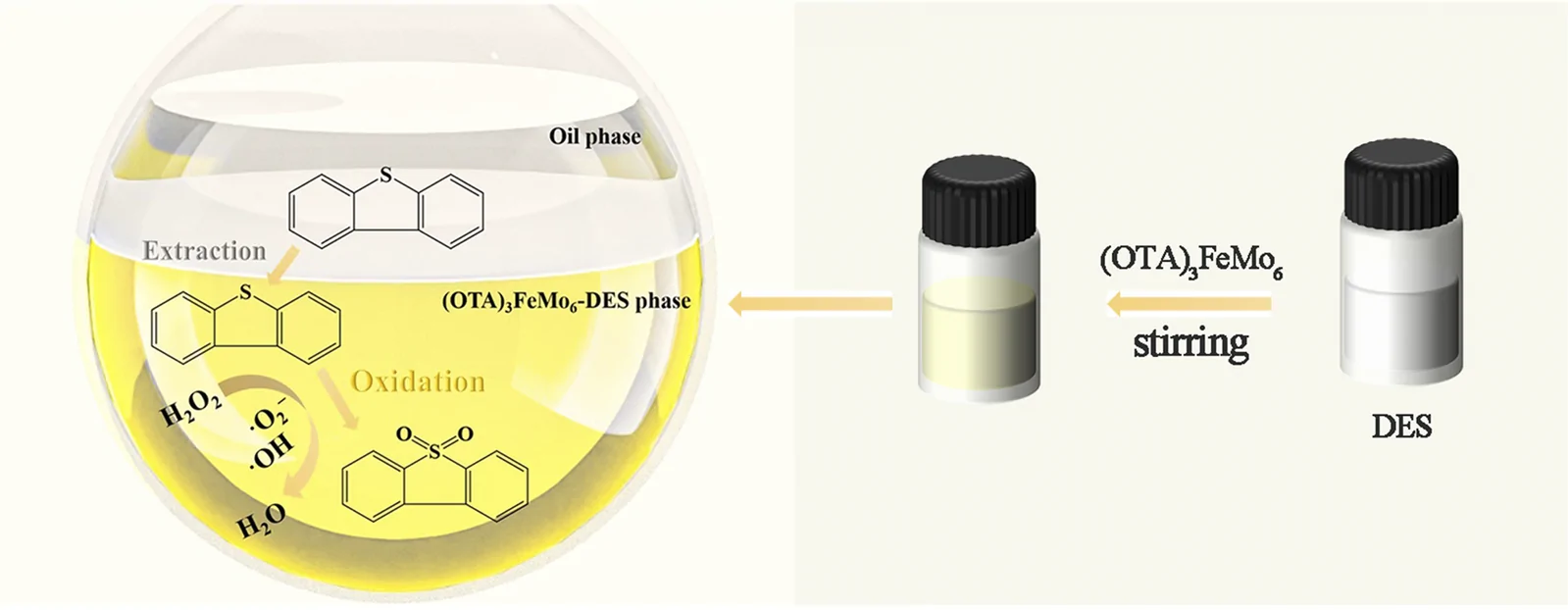

## 1. Introduction

Although new energy vehicles are increasingly being developed, conventional fuel-powered vehicles still dominate the global market.^1^ However, the sulfur compounds present in fuels release toxic and harmful sulfur oxides (SO_*x*_) upon combustion, posing significant risks to human health and contributing to environmental issues such as acid rain.^2,3^ To mitigate SO_*x*_ emissions at the source, governments worldwide have implemented stringent regulations, mandating that sulfur content in fuels must not exceed 10 ppm.^4,5^ Currently, hydrodesulfurization (HDS) is the most widely employed industrial method for removing sulfides from fuel oils. While HDS effectively eliminates simple sulfides (*e.g.*, mercaptans and disulfides), its efficiency in removing refractory sulfur compounds—such as benzothiophene (BT) and dibenzothiophene (DBT), which possess aromatic structures with high steric hindrance, remains limited.^6,7^ Additionally, HDS operates under high-pressure and high-temperature conditions, imposing stringent equipment requirements and increasing production costs. Consequently, there is a pressing need to develop alternative desulfurization technologies.^5,8,9^ In response, several advanced desulfurization methods have emerged, including oxidative desulfurization (ODS),^10^ biological desulfurization (BDS),^11,12^ adsorption desulfurization (ADS),^13^ and photocatalytic oxidative desulfurization (PODS).^14,15^ Among these, ODS has garnered significant attention due to its mild reaction conditions and high efficiency in removing refractory sulfur compounds.^16–18^

ODS is a process that removes sulfur-containing compounds from fuel oil through oxidation. Common oxidants employed in ODS include molecular oxygen (O_2_), hydrogen peroxide (H_2_O_2_), and *tert*-butyl hydroperoxide (TBHP).^19,20^ Among these, H_2_O_2_ is particularly advantageous due to its environmentally benign reduction product (water) and high sustainability, making it one of the most widely used oxidants in ODS systems.^21^ However, the desulfurization efficiency achieved by oxidants alone is typically insufficient for deep desulfurization, necessitating the use of catalysts to enhance performance. The selection of an appropriate catalyst is therefore critical for optimizing the oxidative desulfurization process.^22,23^

Currently, the most widely used catalytic materials in ODS include POMs,^24,25^ metal–organic frameworks (MOFs),^26,27^ and metal oxides.^28–30^ Among these, POMs exhibit exceptional potential in catalytic oxidative desulfurization due to their strong Brønsted acidity, tunable molecular structures, and environmental compatibility.^31–33^ A notable subclass, Anderson-type polyoxometalates, feature a planar hexagonal structure consisting of six edge-sharing MoO_6_ octahedra surrounding a central XO_6_ octahedron (where X = Fe, Co, Ni, *etc.*). This unique architecture, with its two terminal oxygen atoms, confers high catalytic activity in oxidation reactions.^34,35^ However, most POM based catalysts tend to have high reaction temperature, which makes it difficult to achieve room temperature desulfurization. In addition, only catalysts and oxidants can oxidize sulfur-containing compounds in fuel oil into corresponding sulfones or sulfoxides, but cannot separate them from fuel oil. In order to separate sulfide from fuel oil, extractant is usually added to the system to extract oxidation products from fuel oil.

In ODS, conventional extractants include organic solvents (*e.g.*, acetonitrile),^36,37^ ionic liquids (ILs),^38^ and DES.^39^ As ionic liquid analogues, DESs are homogeneous mixtures formed through hydrogen-bonding interactions between hydrogen-bond donors and hydrogen-bond acceptors at specific molar ratios.^40^ In ODS, DES not only possesses extraction capability, but also exhibits certain catalytic properties.^41,42^ Additionally, DESs offer advantages such as biodegradability, simple preparation, and low cost, making them increasingly prominent in desulfurization research.^43,44^

Based on the above analysis, a liquid catalyst POM-DES was successfully prepared by combining Anderson type POM with *p*-toluenesulfonic acid based ternary DES through a facile stirring method. This catalyst achieved the combination of excellent properties of POM and DES, not only showed excellent catalytic activity, but also had good extraction performance. The successful synthesis of the catalyst was confirmed by FT-IR, Raman spectroscopy and ^1^H NMR. The corresponding factors affecting the desulfurization performance of the catalyst (temperature, DES dosage, POM dosage, *etc.*) were systematically investigated. The results showed that the catalyst composed of Anderson type polyoxometalate (OTA)_3_FeMo_6_ and *p*-toluenesulfonic acid based ternary DES showed excellent performance, and could achieve efficient removal of DBT at room temperature. At the same time, the catalyst showed good tolerance to pyridine and xylene, and had certain potential for the removal of sulfide in real fuel. In addition, the catalyst has good cycle stability and can be reused for more than 5 times without deactivation.

## 2. Experiment

### 2.1. Materials

The chemicals involved are of analytical purity and have not been further purified before use. (NH_4_)_6_Mo_7_O_24_·4H_2_O, Fe_2_(SO_4_)_3_, ethanol, deuterated DMSO, octadecyl trimethyl ammonium bromide (OTAB), ZrOCl_2_·8H_2_O, *p*-TsOH, ethylene glycol, dibenzothiophene (DBT), benzothiophene (BT), 4,6-dimethyldibenzothiophene (4,6-DMDBT), biphenyl, *n*-octane, hydrogen peroxide (H_2_O_2_, 30 wt%).

### 2.2. Synthesis of DES

The synthesis was conducted following a previously reported method:^45^ ZrOCl_2_·8H_2_O (0.322 g), *p*-toluenesulfonic acid (1.9 g), and ethylene glycol (580 µL) were sequentially added to a round-bottom flask. The flask was then immersed in an oil bath preheated to 100 °C and stirred continuously for approximately one hour until all solid components had completely dissolved, forming the desired DES. The as-prepared DES was subsequently transferred into a glass vial and stored under sealed conditions.

### 2.3. Synthesis of (OTA)_3_FeMo_6_-DES catalyst

The (OTA)_3_FeMo_6_-DES catalyst was prepared *via* the following procedure. Briefly, 0.02 g of (OTA)_3_FeMo_6_ (synthesis details are provided in the SI) was added into a 5 mL glass vial and mixed with 1 mL of deep eutectic solvent (DES). The mixture was sealed and magnetically stirred at room temperature for 24 h to achieve full homogenization. Finally, a homogeneous catalytic system named (OTA)_3_FeMo_6_-DES was successfully obtained.

### 2.4. Catalyst characterization and performance test

The functional groups and chemical bonds present in the synthesized substances were analyzed using FT-IR spectroscopy (Bio-rad FTS 6000). The types and molar ratios of hydrogen atoms, as well as the intermolecular interactions within the substances, were determined *via*^1^H NMR spectroscopy (AV400, Bruker), employing deuterated DMSO as the solvent. Raman spectroscopy (Horiba HR Evolution) was also utilized to further confirm the functional groups and chemical bonds present in the samples. The viscosity of the substances was measured using a TA HR-10 rotary shear rheometer. Upper oil samples were analyzed using an Agilent 7820A gas chromatography (GC) system equipped with a DB-5 capillary column (25 m × 0.32 nm × 0.5 mm). Detection was performed with a flame ionization detector (FID) under the following conditions: injection volume of 1 µL, column temperature at 150 °C, the front injection temperature is 200 °C, and the back injection temperature is 250 °C. Additionally, oxidation products were identified and analyzed using gas chromatography-mass spectrometry (GC-MS; Agilent 8860 + 5977C).

## 3. Results

### 3.1. Characterization

The FT-IR spectra in Fig. 1a show the characteristic absorption bands of three surface-modified polyoxometalates (POMs). The peaks at 2919 and 2850 cm^−1^ correspond to the C–H bending vibration of the alkyl chain in the surfactant counterion, while the peak at 1467 cm^−1^ represents the C–H bending vibration. The POM framework exhibited unique vibration modes at 551 and 652 cm^−1^ (bridging oxygen bond MO–O–Mo) and 907–948 cm^−1^ (terminal Mo=O extension),^46^ confirming that the POM surfactant successfully formed amphiphilic hybrids with different alkyl chain lengths.^47,48^Fig. 1b shows the FT-IR spectra of the deep eutectic solvent (DES) components and the final DES product. Pure ethylene glycol exhibits typical O–H (3270 cm^−1^) and C–H (2856–2921 cm^−1^) vibrations. The DES spectrum shows a displaced O–H stretch band (3434 cm^−1^), indicating the formation of hydrogen bonds between the components.^45^ The spectra of the POM-DES hybrid system (Fig. 1c) are almost the same as those of pure DES, indicating that the DES structure is retained after POM incorporation. The absence of obvious POM characteristic peaks may be due to the overlap of POM characteristic peaks with DES characteristic peaks or the POM concentration being lower than the detection limit. To this end, we increased the POM content in the catalyst and characterized the samples by infrared spectroscopy, as shown in Fig. 1d. With increasing POM content, characteristic absorption peaks appeared at 919 and 954 cm^−1^, which are attributed to (OTA)_3_FeMo_6_. The rise in POM loading did not induce any shifts or changes in the infrared absorption peaks of other components, confirming the successful incorporation of POM and its compatible coexistence with the other substances in the catalyst.

**Fig. 1:**
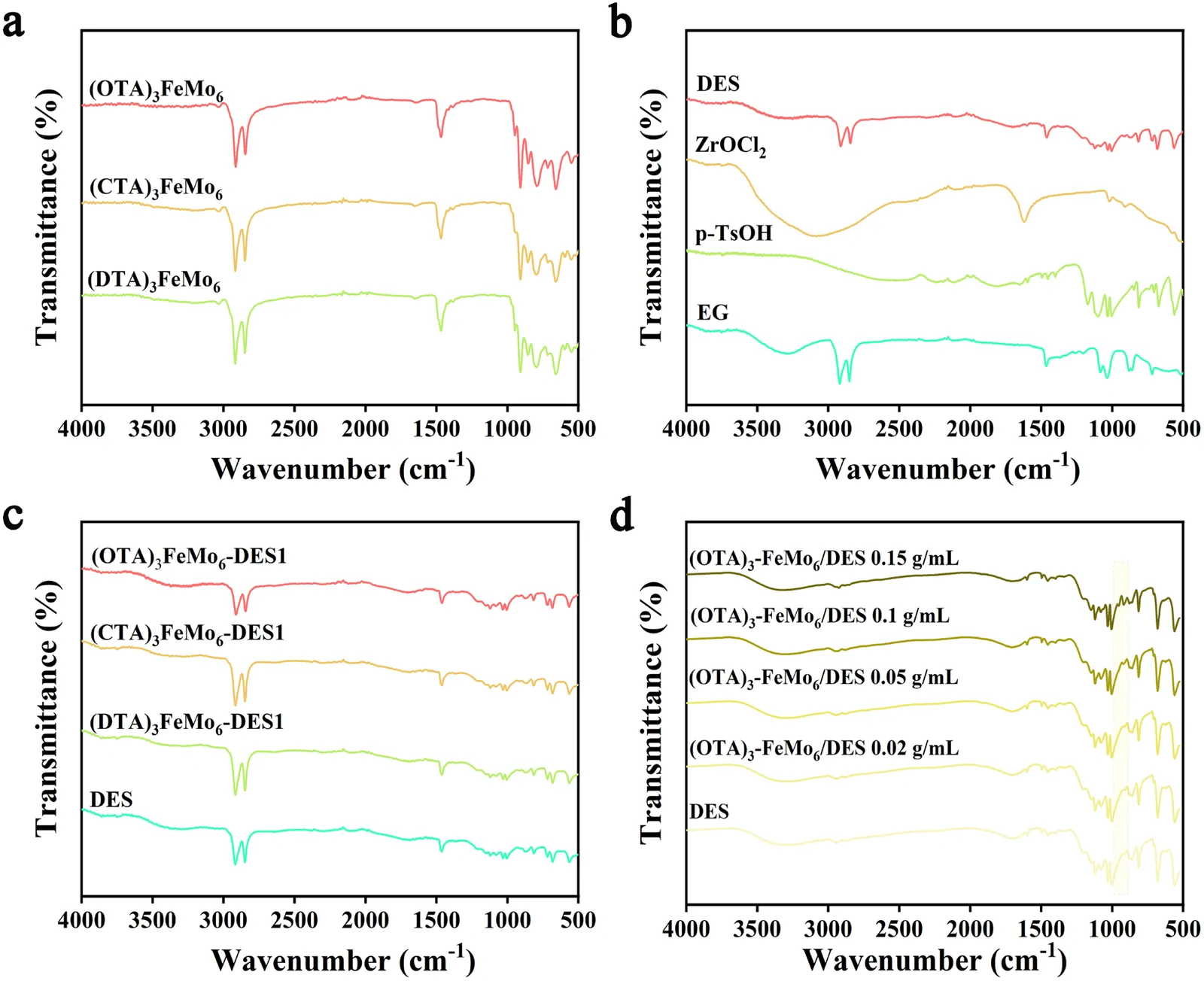
FT-IR spectra of (a) (DTA)_3_FeMo_6_, (CTA)_3_FeMo_6_, (OTA)_3_FeMo_6_, (b) EG, *p*-TsOH, ZrOCl_2_, DES, (c) DES, (DTA)_3_FeMo_6_-DES, (CTA)_3_FeMo_6_-DES, (OTA)_3_FeMo_6_-DES, (d) DES and catalysts with different contents of POM.

To further confirm the presence of POM in the catalyst, Raman spectroscopy characterization was performed, as shown in Fig. 2. The spectrum exhibits characteristic peaks of POM at 900–980 cm^−1^, corresponding to the terminal Mo=O stretching vibrations, and at 576 cm^−1^, attributed to the bridging Mo–O–Mo stretching vibrations. Additionally, the peaks observed at 2800–3000 cm^−1^ are assigned to C–H stretching vibrations from alkyl chains. In the spectrum of pure DES, the absorption peak at 1120 cm^−1^ is ascribed to the –SO_3_H stretching vibration, while the peaks at 2935 and 3069 cm^−1^ correspond to C–H and O–H stretching vibrations from ethylene glycol, respectively. Notably, compared with the Raman spectrum of pure DES, the catalyst spectrum exhibits new characteristic peaks at 959 and 971 cm^−1^, which are attributed to terminal oxygen vibrations of POM. These results provide clear evidence for the successful incorporation of POM into the catalyst system.

**Fig. 2:**
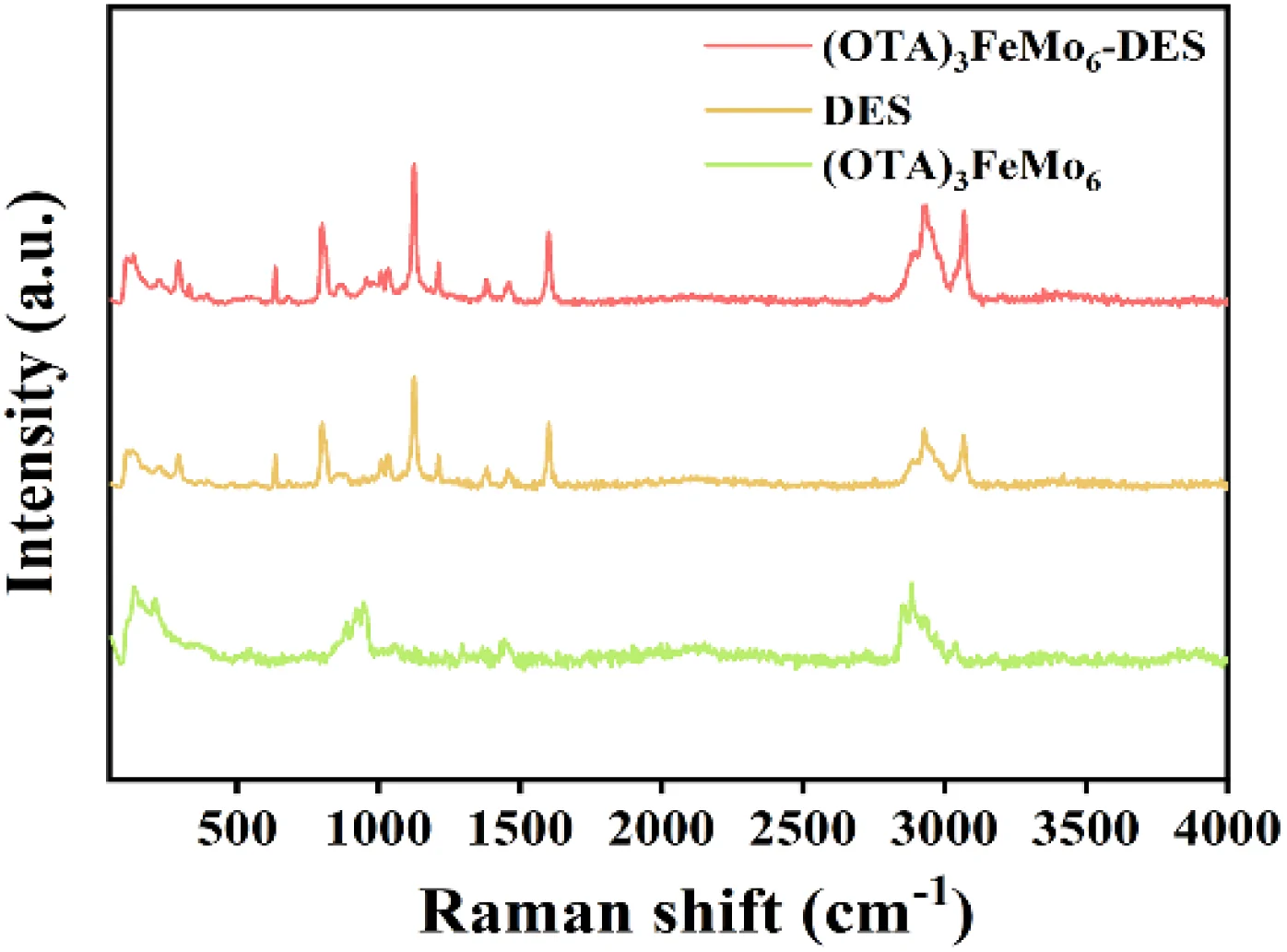
Raman spectra of DES, (OTA)_3_FeMo_6_, (OTA)_3_FeMo_6_-DES.

Fig. 3a and b show the ^1^H NMR spectra of DES and its components as well as three catalysts. In pure DES (Fig. 3a), the characteristic hydroxyl proton resonance of ethylene glycol at 4.46 ppm underwent a significant shift to 5.19 ppm, confirming the formation of hydrogen bonds – a finding consistent with the FT-IR observations.^45^ Introducing POMs into DES (Fig. 3b) will cause further chemical shift disturbances in the proton signal of DES, especially hydroxyl resonance at 5.19 ppm. This phenomenon might be caused by the paramagnetic effect of the iron centers in the POM framework, which provides direct evidence for the incorporation of POM into the hybrid system. The absence of obvious POM signals in all mixed solutions can be attributed to the low POM concentration, which is consistent with the FT-IR spectral observation results.

**Fig. 3:**
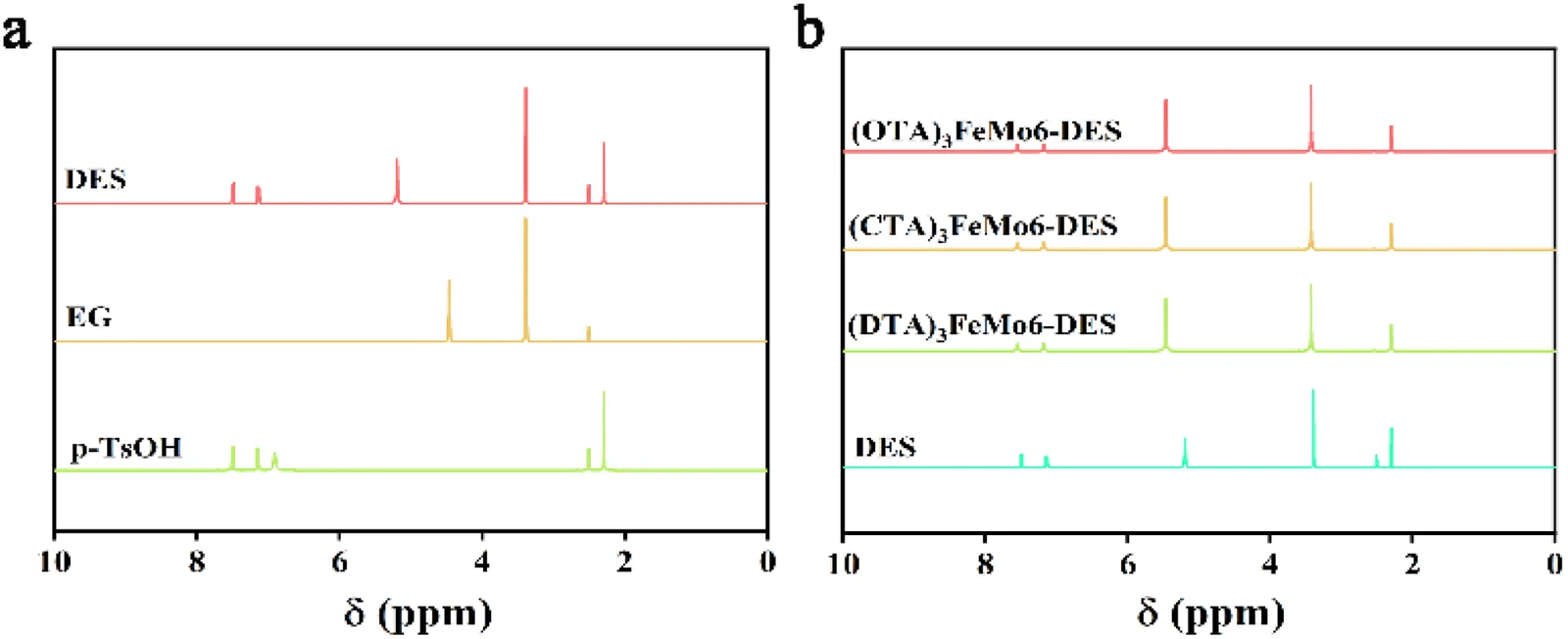
^1^H NMR of (a) *p*-TsOH, EG, DES, (b) DES, (DTA)_3_FeMo_6_-DES, (CTA)_3_FeMo_6_-DES, (OTA)_3_FeMo_6_-DES.

To further investigate the correlation between viscosity and desulfurization performance, the viscosity of the catalysts was systematically measured. As shown in Fig. 4, the incorporation of POM and the elongation of alkyl chains were found to increase the catalyst viscosity, which exhibited a positive correlation with the oxidative desulfurization performance: (OTA)_3_FeMo_6_-DES > (CTA)_3_FeMo_6_-DES > (DTA)_3_FeMo_6_-DES > DES. This phenomenon can be attributed to the fact that a moderate increase in viscosity enhances the extraction efficiency, thereby improving desulfurization performance.^5^ Additionally, the viscosity of the catalyst gradually decreased with increasing temperature, which aligns with the observed temperature-dependent desulfurization behavior (where performance initially increases and then decreases with rising temperature). At excessively low temperatures, the high viscosity increases mass transfer resistance, resulting in limited desulfurization efficiency. Conversely, excessively high temperatures reduce viscosity, diminishing the extraction capability and consequently lowering desulfurization performance.^49,50^

**Fig. 4:**
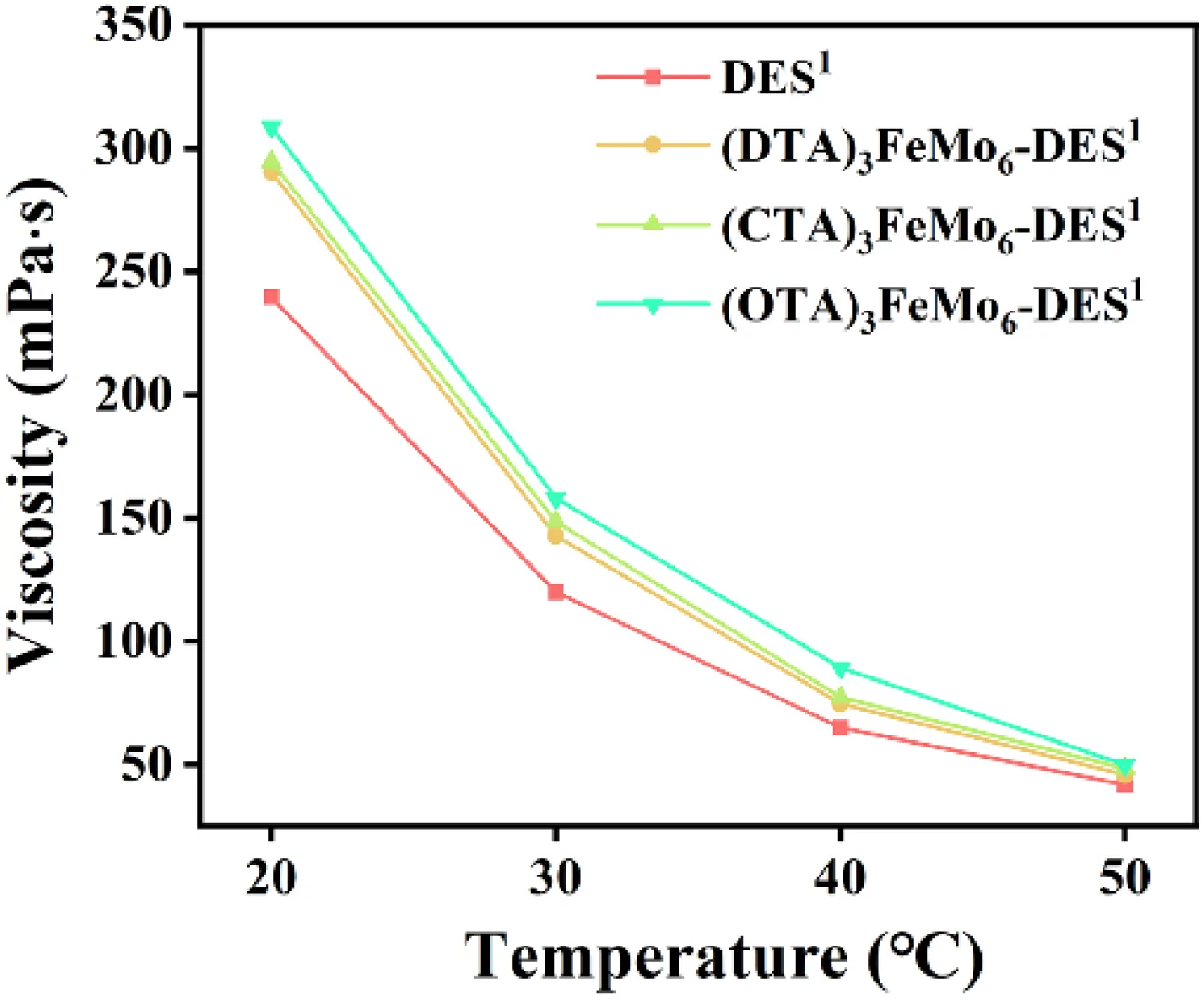
Viscosity of each substance as a function of temperature.

### 3.2. Desulfurization performance analysis

To demonstrate that the mixture of POM and DES can enhance the catalytic performance of a single type of substance to a certain extent, we systematically evaluated the desulfurization efficiency of different catalytic systems under the same reaction conditions. As shown in Table 1, the POM/H_2_O_2_ catalytic oxidation system alone only exhibited limited activity, with a conversion rate of DBT of only 28.6%, while the DES/H_2_O_2_ extraction catalytic oxidation system showed a moderate improvement (DBT removal rate of 60.8%). Remarkably, the integrated POM-DES/H_2_O_2_ hybrid system achieved complete elimination of DBT under the same conditions. These comparative results clearly indicate that the synergistic combination of POM and DES is an effective strategy for achieving deep desulfurization under mild operating conditions.

**Table 1:** Influence of different catalytic systems on the DBT removal efficiency

Entry	System	Efficiency
1	H_2_O_2_ (16.5 µL)	6.6%
2	H_2_O_2_ (16.5 µL) + DES (1 mL)	60.80%
3	(OTA)_3_FeMo_6_ (0.02 g) + H_2_O_2_ (16.5 µL)	28.60%
4	(OTA)_3_FeMo_6_-DES (POM: 0.02 g, DES: 1 mL) + H_2_O_2_ (16.5 µL)	100%

The influence of alkyl chain length in POMs on desulfurization efficiency was systematically investigated. As demonstrated in Fig. 5a, catalytic activity exhibited a clear dependence on chain length, with performance improving as the alkyl chains became longer. Specifically, (OTA)_3_FeMo_6_-DES achieved complete DBT removal within 25 minutes, while (CTA)_3_FeMo_6_-DES required 30 minutes for full conversion. In contrast, (DTA)_3_FeMo_6_-DES showed limited performance, removing only 96% of DBT within the same time. This trend suggests that longer alkyl chains enhance POM hydrophobicity, thereby improving both DBT extraction efficiency and interfacial contact between the catalyst and substrate, ultimately leading to superior desulfurization performance.^51,52^ A good linear relationship was obtained when plotting ln(*C*_0_/*C*_*t*_) *versus* reaction time *t*, as shown in the updated Fig. 5b. These results clearly indicate that the catalytic reaction follows a pseudo-first-order kinetic model.

**Fig. 5:**
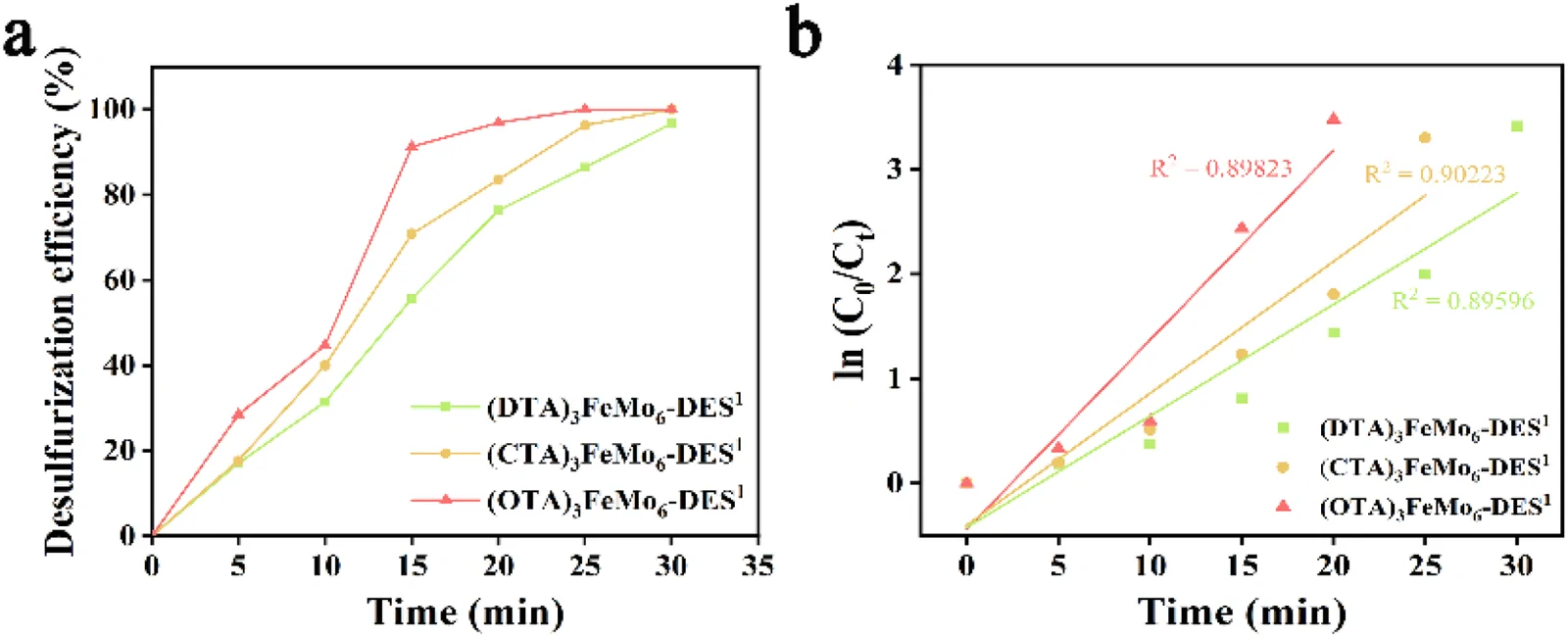
(a) Influence of alkyl chain length on DBT removal rate, (b) kinetic equation.

Temperature also plays a crucial role in influencing the desulfurization performance of catalysts. To this end, this study explored the desulfurization performance of the catalyst at temperatures of 20 °C, 30 °C, 40 °C, and 50 °C. Fig. 6 illustrates that at 20 °C, the catalyst's desulfurization effect was not pronounced, with only 85% of DBT removed within 30 minutes. As the temperature increased to 30 °C, the catalyst's desulfurization efficiency improved, enabling the complete removal of DBT within 25 minutes. However, as the temperature continued to rise, there was little change in the catalyst's desulfurization efficiency. This may be attributed to the impact of temperature on the catalyst's viscosity and the enhanced decomposition of hydrogen peroxide, which subsequently affects its desulfurization performance.^44,53,54^ Consequently, even with an increase in temperature, there was no significant change in the catalyst's desulfurization capabilities.

**Fig. 6:**
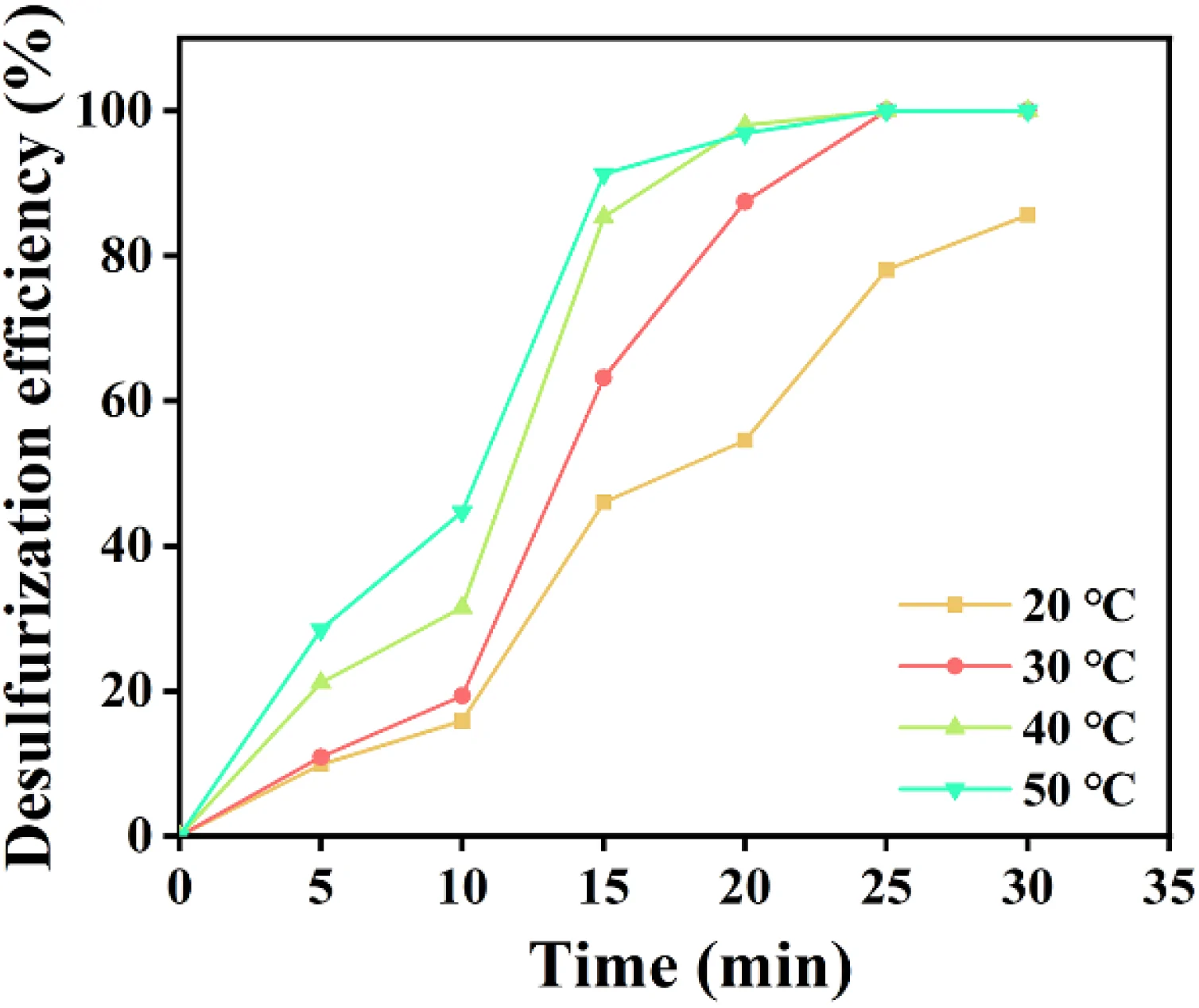
Influence of temperature on DBT removal rate.

Fig. 7 demonstrates the influence of catalyst dosage on desulfurization efficiency. The system achieved complete DBT removal within 30 minutes at 0.5 mL catalyst dosage, while increasing the dosage to 1 mL reduced the required time to 25 minutes. Notably, further dosage elevation to 1.5 mL provides no significant improvement in desulfurization efficiency despite maintaining 100% DBT conversion for 25 minutes, likely due to the dilution effect of H_2_O_2_ caused by the increase of catalyst dosage.^55^ These results establish 0.5 mL as the optimal catalyst dosage when considering both performance and economic feasibility.

**Fig. 7:**
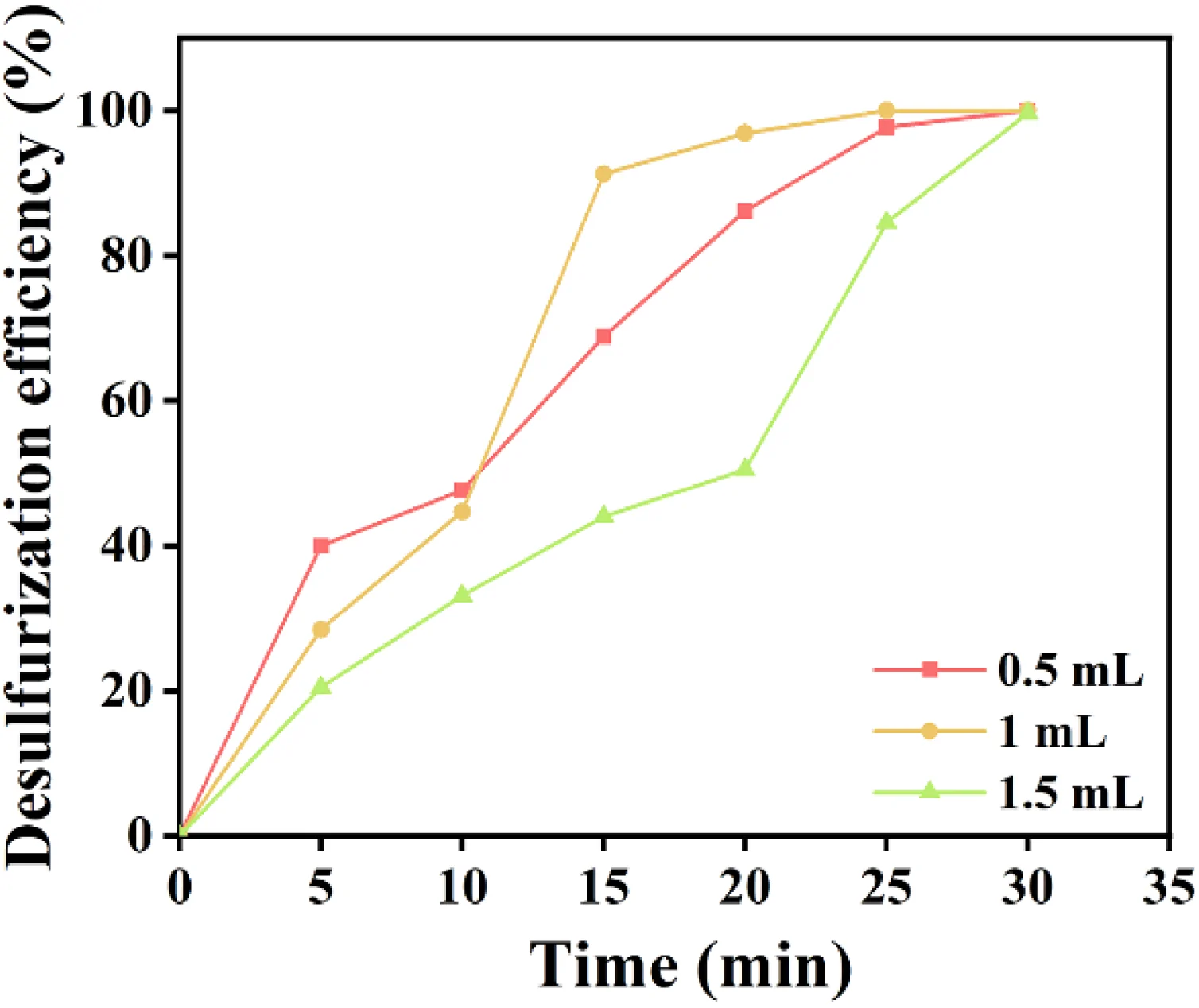
Influence of catalyst dosage on DBT removal rate.

The dosage of oxidant in oxidative desulfurization is also a factor worthy of attention. Theoretically, when the molar ratio of the oxidant to DBT is 2 (O/S = 2), DBT can be completely oxidized to the corresponding sulfone.^1,56^ However, in this work (Fig. 8), when O/S = 2, only approximately 84% of the DBT was removed within 30 minutes, which might be due to the inevitable decomposition of hydrogen peroxide. By increasing the O/S to 2.8, all DBT can be removed within 30 minutes. If the O/S ratio is further increased to 3, the desulfurization efficiency does not increase significantly and it still takes 30 minutes to completely remove the DBT. When O/S = 4, the desulfurization performance of the catalyst slightly decreases, with a 98.59% removal of DBT for 30 minutes. This might due to the increase in the amount of H_2_O_2_ will increase the generation of H_2_O, thereby having a certain dilution effect on the system and leading to a decline in desulfurization performance.^26^ From the above, it can be seen that the optimal O/S is 2.8.

**Fig. 8:**
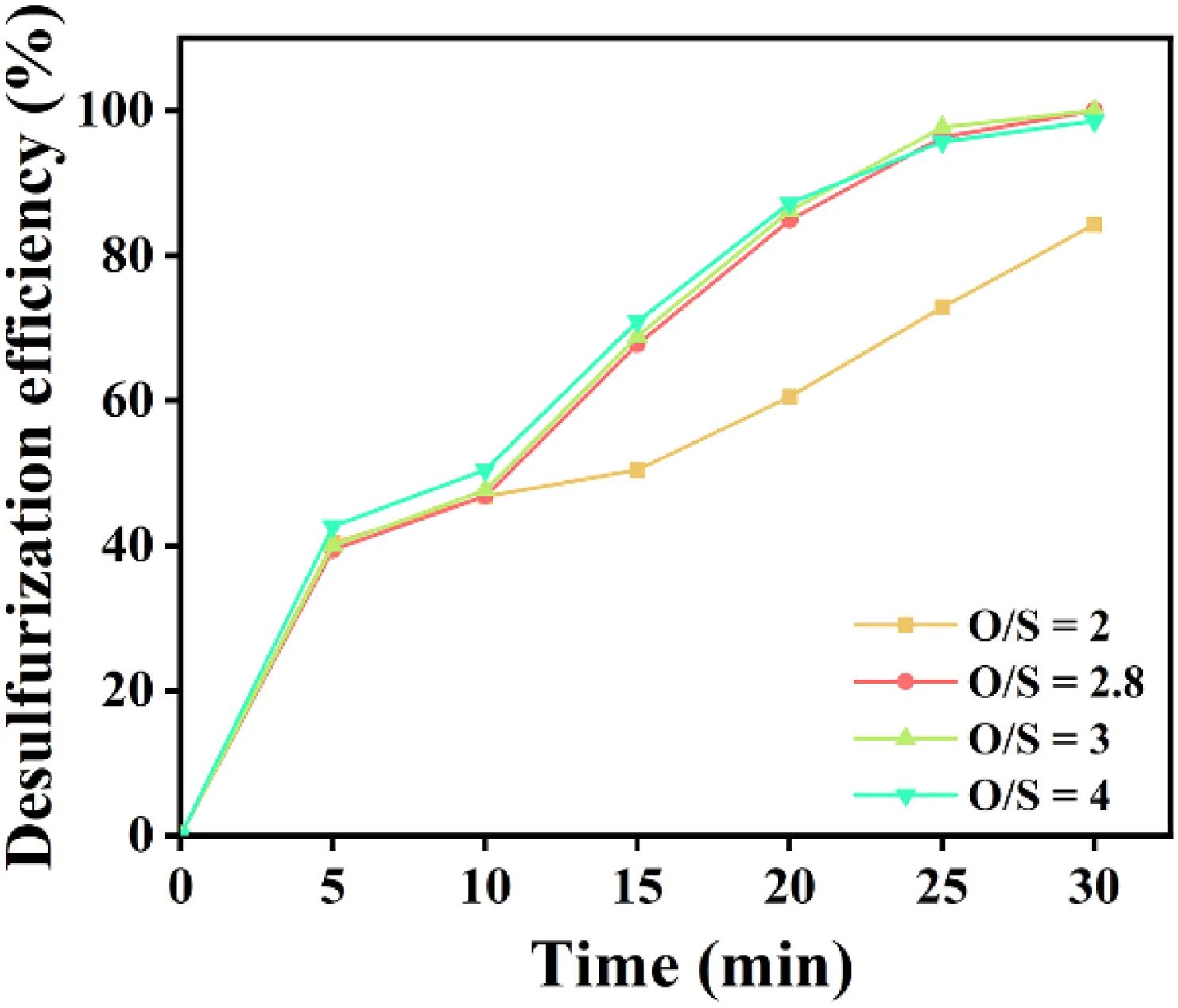
Influence of O/S on DBT removal rate.

The removal of thiophenic sulfur compounds such as BT, and 4,6-DMDBT is a critical metric for evaluating the performance of catalytic oxidative desulfurization systems. As depicted in Fig. 9, the catalytic system exhibits varying removal efficiencies for these sulfur-containing substrates, following the order DBT > 4,6-DMDBT > BT. This trend can be attributed to differences in electron density distribution on sulfur atoms and steric hindrance effects among the substrates. Previous studies indicate that the sulfur atom electron densities follow the sequence 4,6-DMDBT > DBT > BT,^1,57^ which explains the superior removal efficiency of 4,6-DMDBT compared to BT. However, despite its higher electron density, 4,6-DMDBT demonstrates lower removal efficiency than DBT due to increased steric hindrance from its methyl substituents, which impedes effective contact between the catalyst and the sulfur atom.

**Fig. 9:**
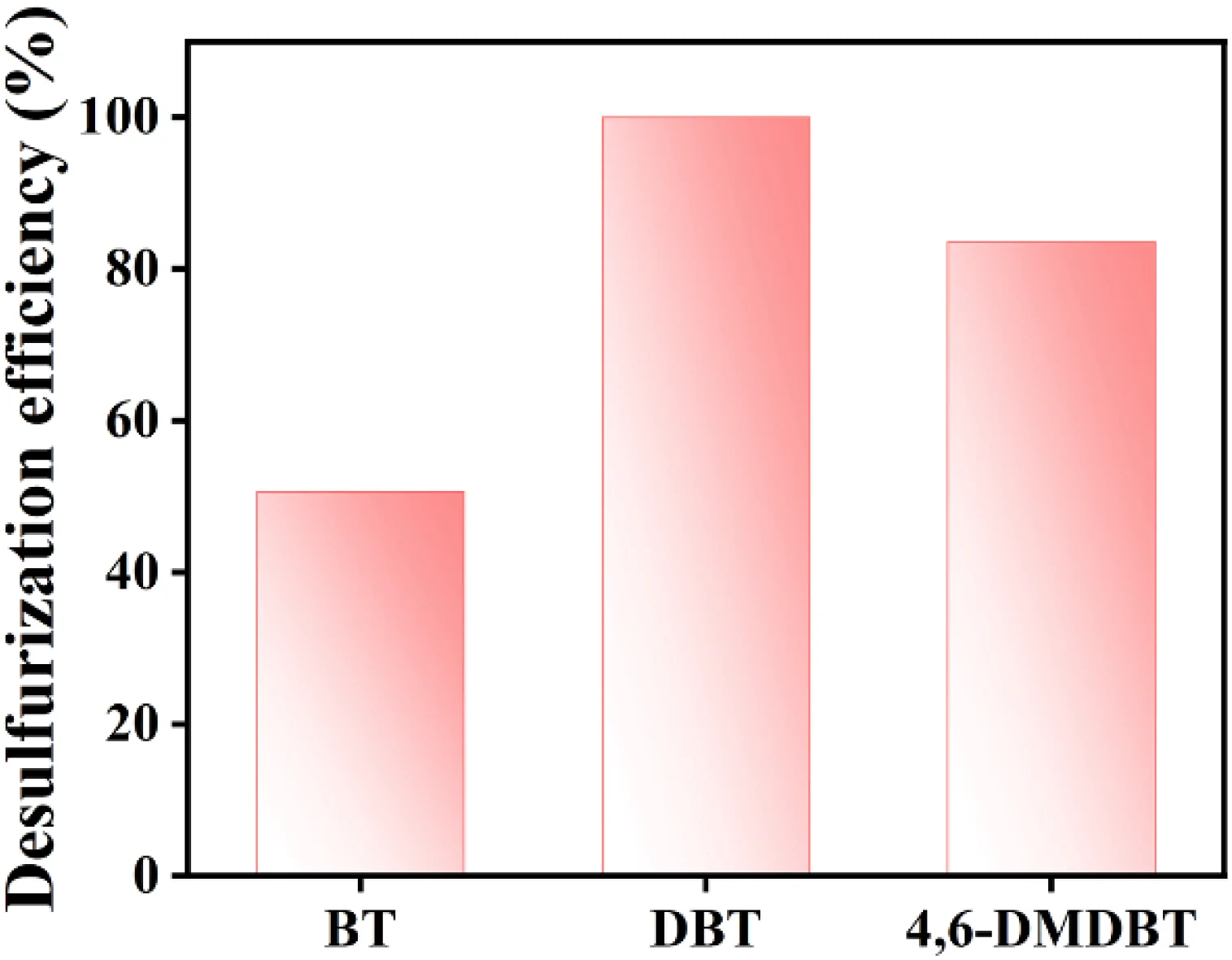
Removal efficiency of BT, DBT and 4,6-DMDBT.

In practical fuel oils, aromatic compounds and nitrogen-containing species are commonly present, which can significantly influence the desulfurization performance of catalysts.^58^ To assess the applicability of this catalyst for real fuel oil treatment, xylene and pyridine were individually introduced into the model oil system to investigate the effects of varying concentrations of aromatic and nitrogenous compounds on catalytic desulfurization efficiency. As shown in Fig. S1a, when the concentration of xylene was 1000 µg mL^−1^, the desulfurization performance remained stable. Further increasing the xylene content to 10 000 µg mL^−1^ resulted in a DBT removal rate exceeding 90%. However, at a xylene concentration of 15 000 µg mL^−1^, a notable decline in catalytic performance was observed. The impact of pyridine on desulfurization was even more pronounced (Fig. S1b). With increasing pyridine levels, the desulfurization efficiency gradually diminished. Specifically, when the pyridine concentration reached 5000 µg mL^−1^, the DBT removal rate was 93.55%, but further increasing the pyridine concentration to 10 000 µg mL^−1^ led to a reduction in removal efficiency to 83.35%. Overall, the catalyst demonstrates excellent sulfur removal capability even in the presence of moderate amounts of aromatic and nitrogen-containing compounds. Therefore, it holds promising potential for the desulfurization of real fuel oils.

To evaluate the cycling stability performance of the catalytic system, the upper oil phase was separated by decantation after each oxidation desulfurization experiment, following which fresh model oil and H_2_O_2_ were introduced to conduct subsequent cyclic tests. The experimental results (Fig. 10) demonstrate that after five consecutive cycles, the catalytic system still maintains a DBT removal efficiency of 90.76%. The minor decrease in desulfurization performance may be attributed to two potential factors: first, the gradual accumulation of sulfur-containing oxidation products at the catalyst's active sites; second, the dilution effect caused by water generated during the reaction on the DES.^23,59^ Furthermore, FT-IR characterization results (Fig. S2) support the structural stability of the catalyst, as the characteristic infrared absorption peaks remain largely unchanged before and after the cycling process. Additionally, thermal filtration experiments (Fig. S3) indicate that the desulfurization efficiency remains nearly constant even after separating the model oil from the catalyst during the reaction, suggesting no significant leaching of active components and excellent structural integrity. Collectively, these findings confirm the outstanding recyclability and stability of the catalytic system.

**Fig. 10:**
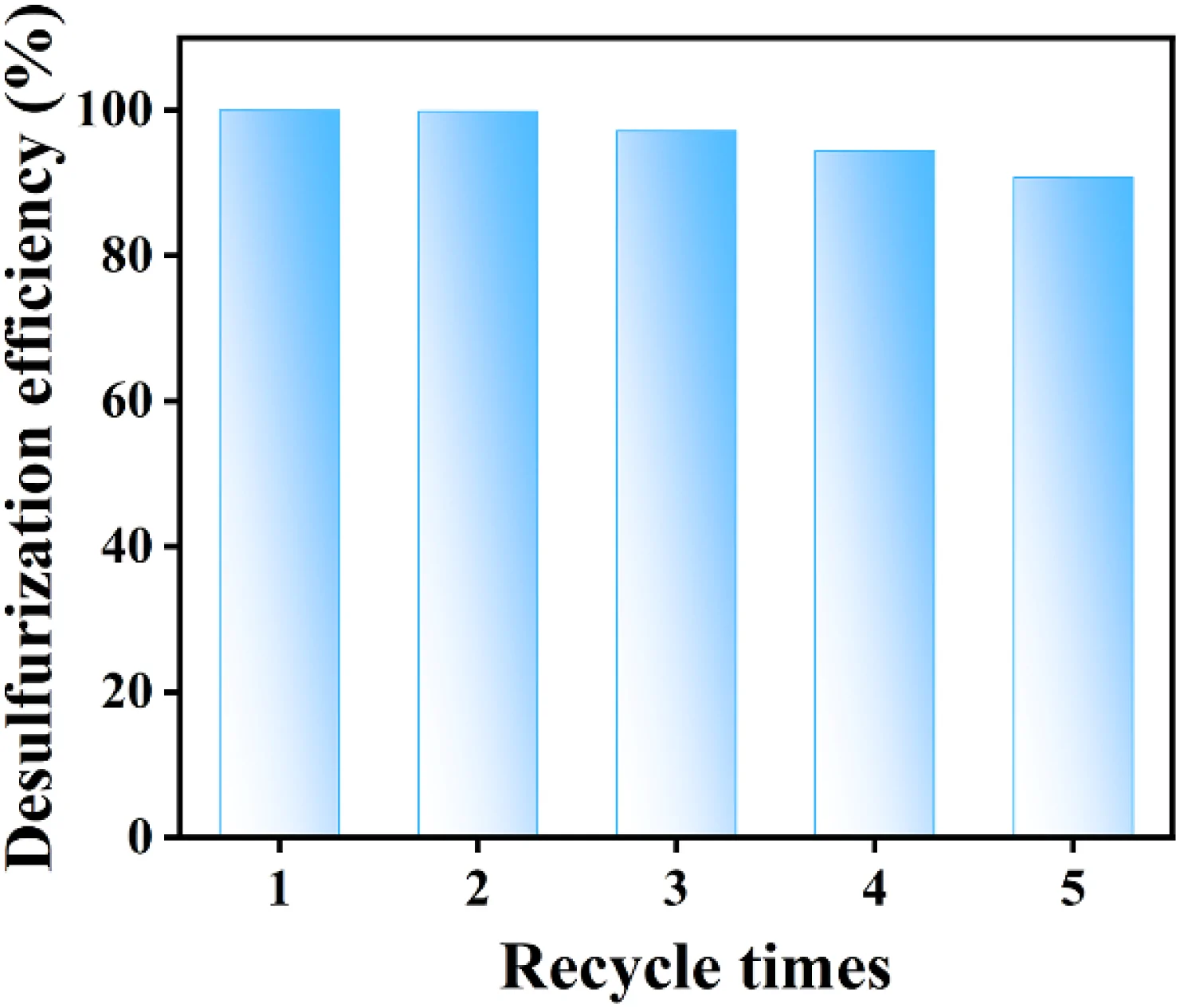
Circulation behavior.

To investigate the oxidation desulfurization mechanism of the catalytic system, this study conducted a systematic analysis by integrating free radical scavenging experiments with product characterization. Initially, isopropyl alcohol (IPA) was employed as a scavenger for hydroxyl radicals (·OH), while *p*-benzoquinone (*p*-BQ) was selected as a scavenger for superoxide radicals (·O_2_^−^). As illustrated in Fig. 11, compared to the control experiment without any scavenger, the addition of either IPA or *p*-BQ resulted in a reduction in desulfurization efficiency. Notably, the inhibitory effect of *p*-BQ was more pronounced, with the desulfurization efficiency decreasing from 100% to 27.94%. This indicates that ·O_2_^−^ plays a dominant role among the reactive oxygen species involved in the oxidation desulfurization process. Subsequently, gas chromatography-mass spectrometry (GC-MS) was employed to analyze the reaction products (Fig. S4a). The results showed that the characteristic peak of dibenzothiophene (DBT) at a retention time of 12.019 min completely disappeared after the reaction, confirming the complete removal of DBT from the model oil. Further analysis of the CCl_4_ extraction phase (Fig. S4b) revealed a new peak at a retention time of 14.548 min, which was identified *via* mass spectrometry as dibenzothiophene dioxide (DBTO_2_, *m*/*z* = 216) (Fig. S5). This confirms that DBT was oxidized into the corresponding sulfone derivative.

**Fig. 11:**
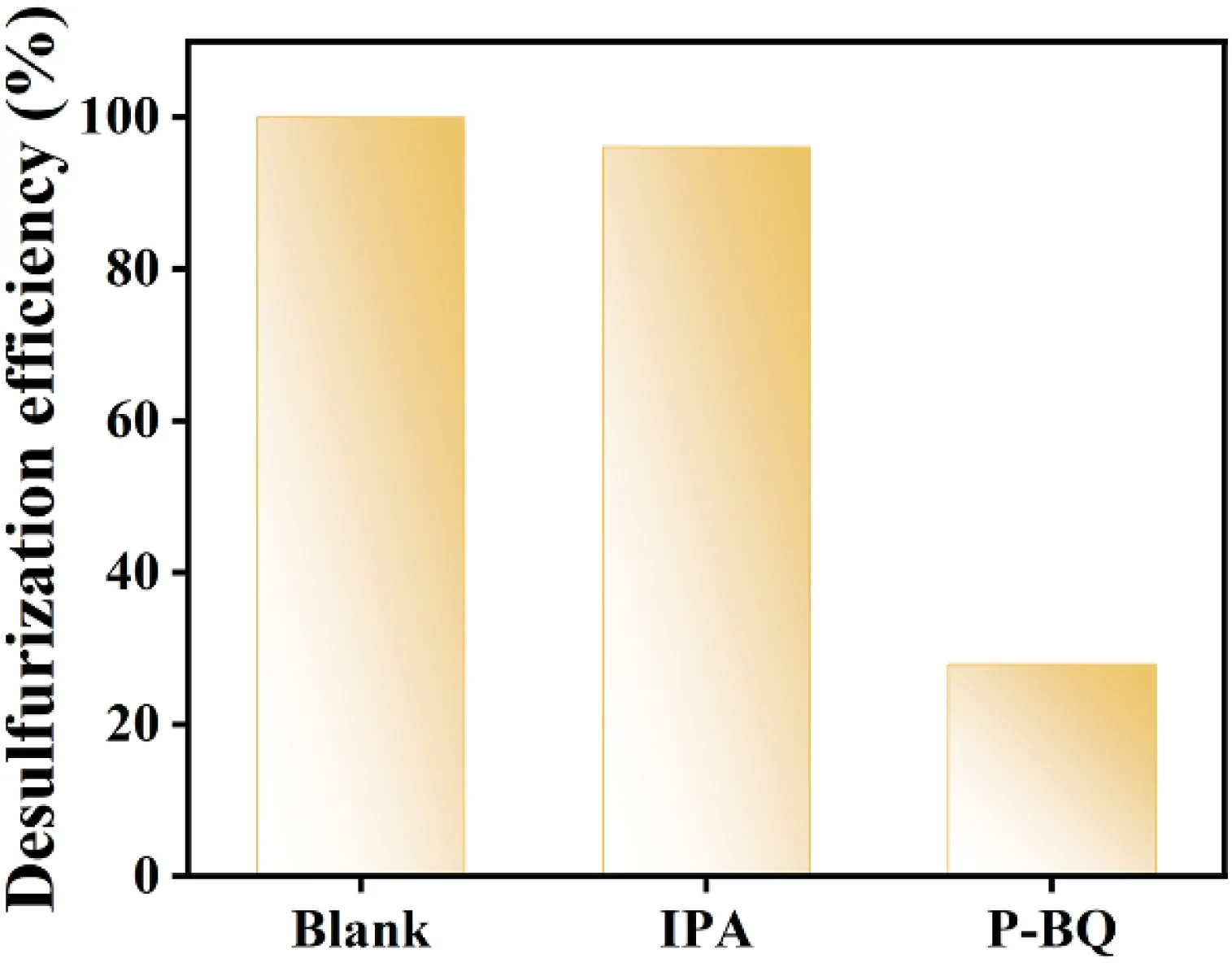
Free radical capture experiment.

Based on the aforementioned experimental findings and analyses, a plausible catalytic reaction mechanism was proposed (as shown in Fig. 12). First, DBT molecules present in the model oil were selectively extracted into the DES phase. Subsequently, H_2_O_2_ generated both O_2_^−^ and ·OH under catalytic conditions. These reactive species then oxidized the DBT transferred into the DES phase to form DBTO_2_, thereby achieving effective removal of sulfur-containing compounds from the fuel.^10^ This mechanism elucidates how the catalytic system accomplishes deep desulfurization through the synergistic effects of “extraction–oxidation,” wherein the formation of peroxide intermediates and their selective oxidation of sulfur compounds are critical steps influencing the overall desulfurization efficiency.

**Fig. 12:**
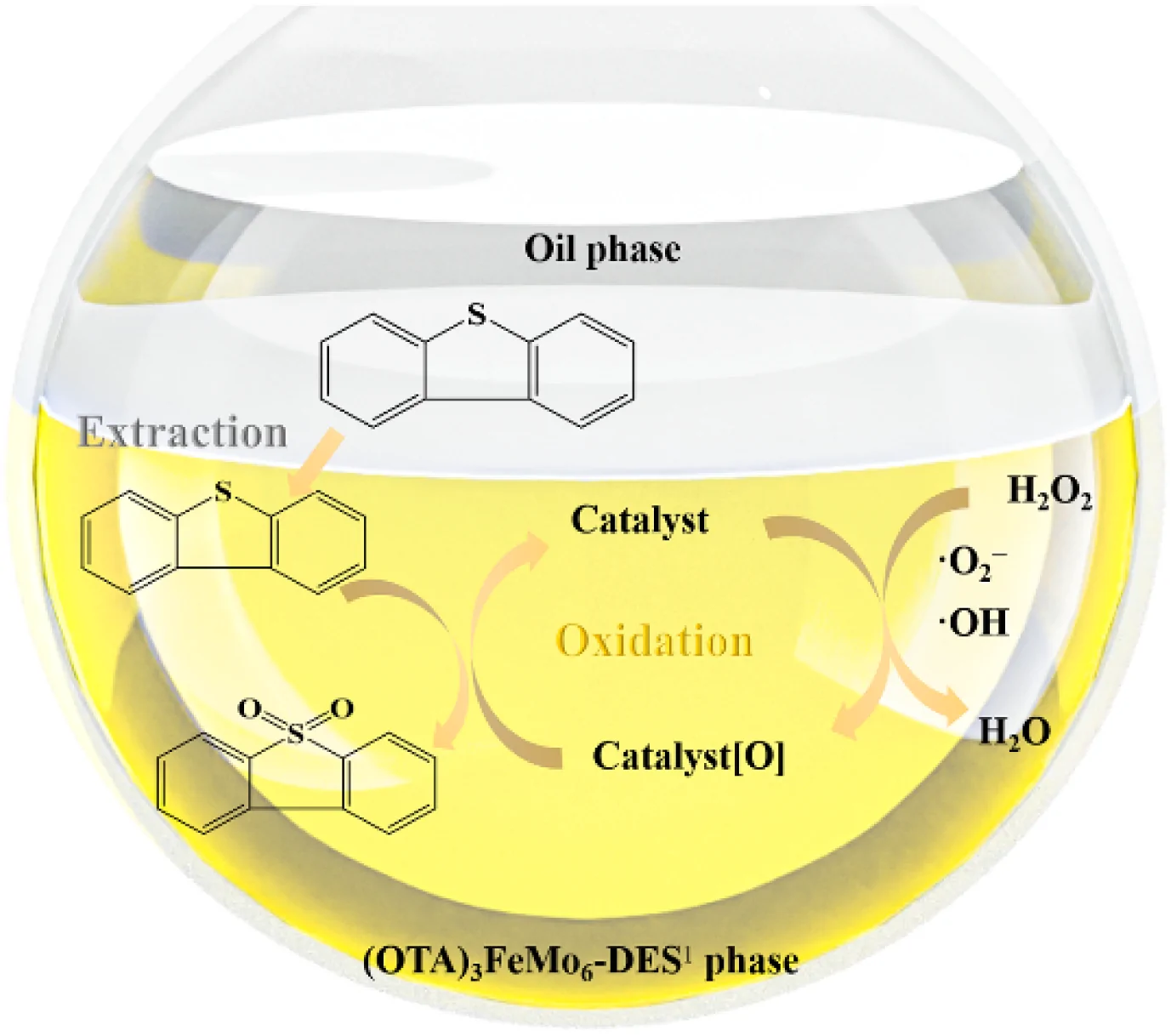
The possible oxidative desulfurization reaction mechanism of (OTA)_3_FeMo_6_-DES.

## 4. Conclusions

In this study, a liquid catalyst, (OTA)_3_FeMo_6_-DES, was successfully synthesized through a synergistic strategy combining POM and DES co-catalysis. The successful formation of the catalyst was confirmed by various characterization techniques, including FT-IR and ^1^H NMR spectroscopy. The factors influencing the catalytic desulfurization performance were systematically investigated, revealing that the catalyst achieves complete removal (100%) of dibenzothiophene (DBT) under optimal conditions (30 °C, O/S = 2.8, DES dosage = 0.5 mL, POM loading = 0.02 g mL^−1^). Moreover, the catalyst exhibited excellent removal efficiency for BT and 4,6-DMDBT. Notably, it maintained high DBT removal efficiency even in model oils containing competitive aromatic compounds such as xylene and pyridine, demonstrating its potential for real fuel desulfurization applications. The desulfurization mechanism was elucidated through radical trapping experiments and GC-MS analysis. Furthermore, recycling tests confirmed the catalyst's excellent stability, as it retained high activity over five consecutive cycles without significant deactivation. These results highlight the promising practical applicability of the (OTA)_3_FeMo_6_-DES catalyst in oxidative desulfurization processes.

## Conflicts of interest

The authors declare no conflicts of interest.

## Supplementary Material

RA-OLF-D6RA02289H-s001

## Data Availability

The data are available upon request from the author. Supplementary information (SI) is available. See DOI: https://doi.org/10.1039/d6ra02289h.
