# Supplementary material for: (OTA)3FeMo6-DES: a liquid catalyst integrating catalytic and extractive functions for room-temperature oxidative desulfurization
Source: RSC Adv. 2026 Nov 11. Online ahead of print. doi: 10.1039/d6ra02289h (PMC13754043; doi:10.1039/d6ra02289h)
Supplement: Supplementary file 1 [file RA-OLF-D6RA02289H-s001.pdf]

Supplementary Information (SI) for RSC Advances.
This journal is © The Royal Society of Chemistry

$(OTA)_3FeMo_6$-DES: A Liquid Catalyst Integrating Catalytic and Extractive Functions for Room-Temperature Oxidative Desulfurization

Yunying Bai, Yang Hua, Yuan Shen, Xin Meng, Yufan Yang, Hong Zhang *

Key Laboratory of Polyoxometalate and Reticular Material Chemistry of Ministry of Education, Department of Chemistry, Northeast Normal University, Changchun, Jilin 130024, PR China

Email Adress: hope20130122@163.com;

orcid.org/0000-0001-8153-1425

1. Synthesis of $(NH_4)_3FeMo_6O_{40}\cdot nH_2O$

The polyoxometalate was synthesized following a modified literature method.[1] First, 2.5 g of $(NH_4)_6Mo_7O_{24}\cdot 4H_2O$ was dissolved in 40 mL of boiling distilled water to form Solution 1. Separately, 0.5 g of $Fe_2(SO_4)_3$ was dissolved in 29 mL of distilled water to prepare Solution 2. Solution 2 was then added dropwise to Solution 1 under continuous stirring, and the mixture was maintained at boiling temperature with constant stirring for 15 minutes. The resulting solution was concentrated using a hot water bath and subsequently filtered while hot. After cooling the filtrate to room temperature, the supernatant was decanted and the precipitate was washed three times with distilled water. The solid product was then dried overnight in an oven at 60°C, yielding a light yellow powder designated as $FeMo_6$.

2. Synthesis of $(OTA)_3FeMo_6$

The $(OTA)_3FeMo_6$ catalyst was synthesized according to a modified literature procedure.[2] In a typical synthesis, 0.1 g of FeMo6 was first dissolved in 20 mL of distilled water under ambient conditions. Subsequently, 20 mL of an ethanol solution containing 0.0869 g (0.2 mmol) of OTAB was added to the aqueous FeMo6 solution.

The resulting mixture was vigorously stirred at room temperature for 2 hours to ensure complete reaction. The solid product was then collected by filtration and thoroughly washed with distilled water to remove any unreacted precursors and residual solvents. Finally, the obtained material was dried in an oven at 60°C for 24 hours to yield the $(OTA)_3FeMo_6$.

3. Model Oil Preparation and Oxidative Desulfurization Procedure

Dissolve 0.2 g of DBT and 0.1 g of biphenyl (internal standard) in 100 mL of n-octane, and stir continuously until completely dissolved to prepare model oil with a sulfur content of 500 ppm. In the desulfurization reaction, 5 mL of model oil is placed in a 25 mL round-bottom flask and preheated in a temperature-controlled oil bath for 10 to 15 minutes to activate the oil sample. Then, a certain amount of catalyst and hydrogen peroxide oxidant are added in sequence to trigger the reaction. Immediately after adding the oxidant, the initial reference sample (t=0) was collected. Then, the upper oil sample was taken at the same time intervals for quantitative analysis by gas chromatography to determine the sulfur content in the model oil. The calculation formula for the desulfurization efficiency R is $R = (1-C_t/C_0)\times100\%$, where C_0 is the initial

concentration of DBT (500 ppm), and C_t is the residual concentration of DBT at time t.

4. Desulfurization performance analysis

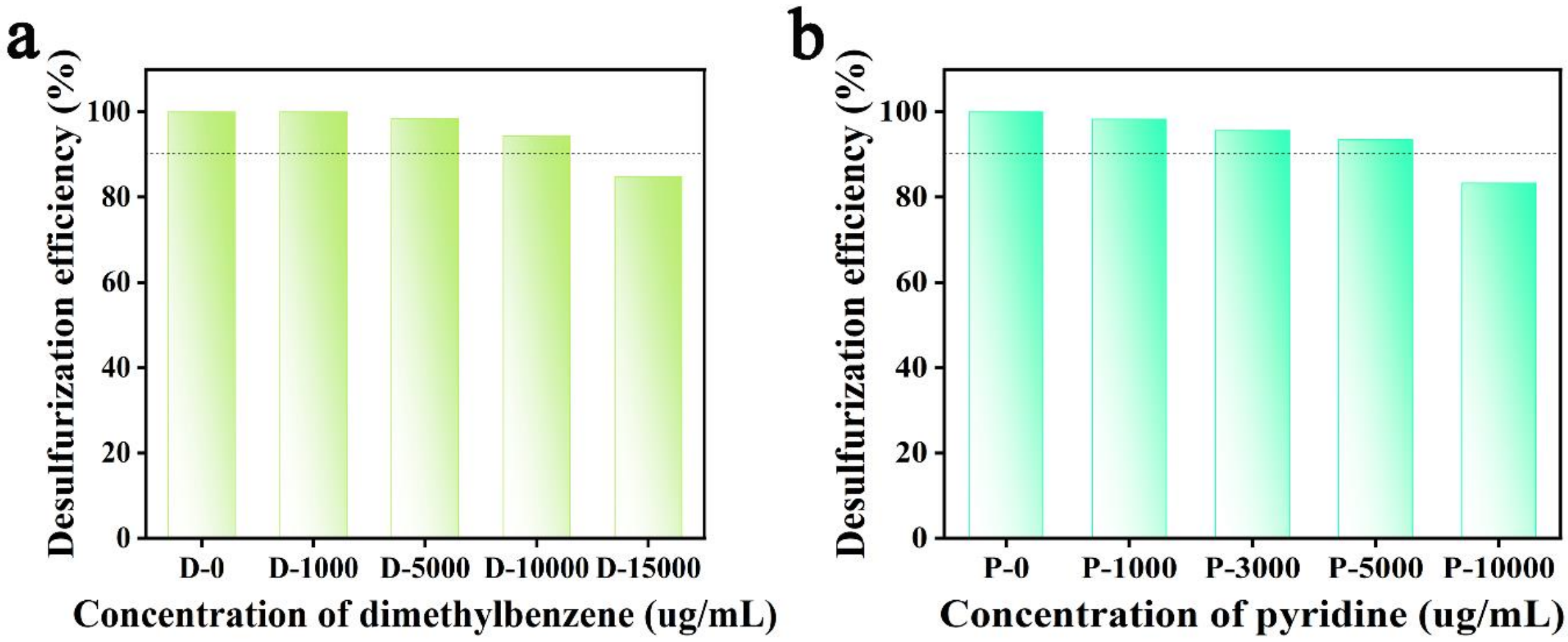

Figure. S1 The effect of (a) dimethybenzene and (b) pyridine on the DBT removal.

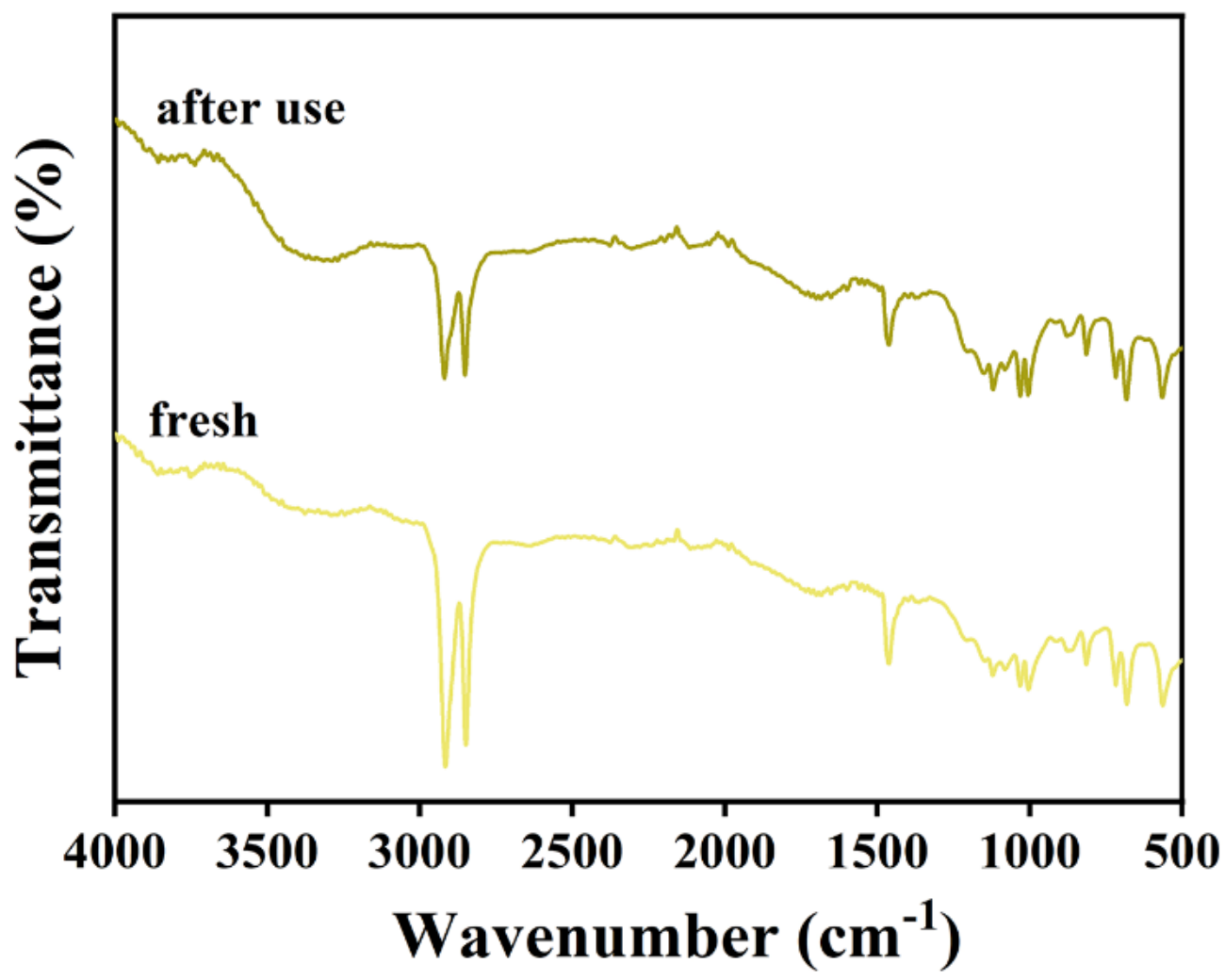

Figure. S2 FT-IR spectra of $(OTA)_3FeMo_6$-DES catalyst before and after reaction

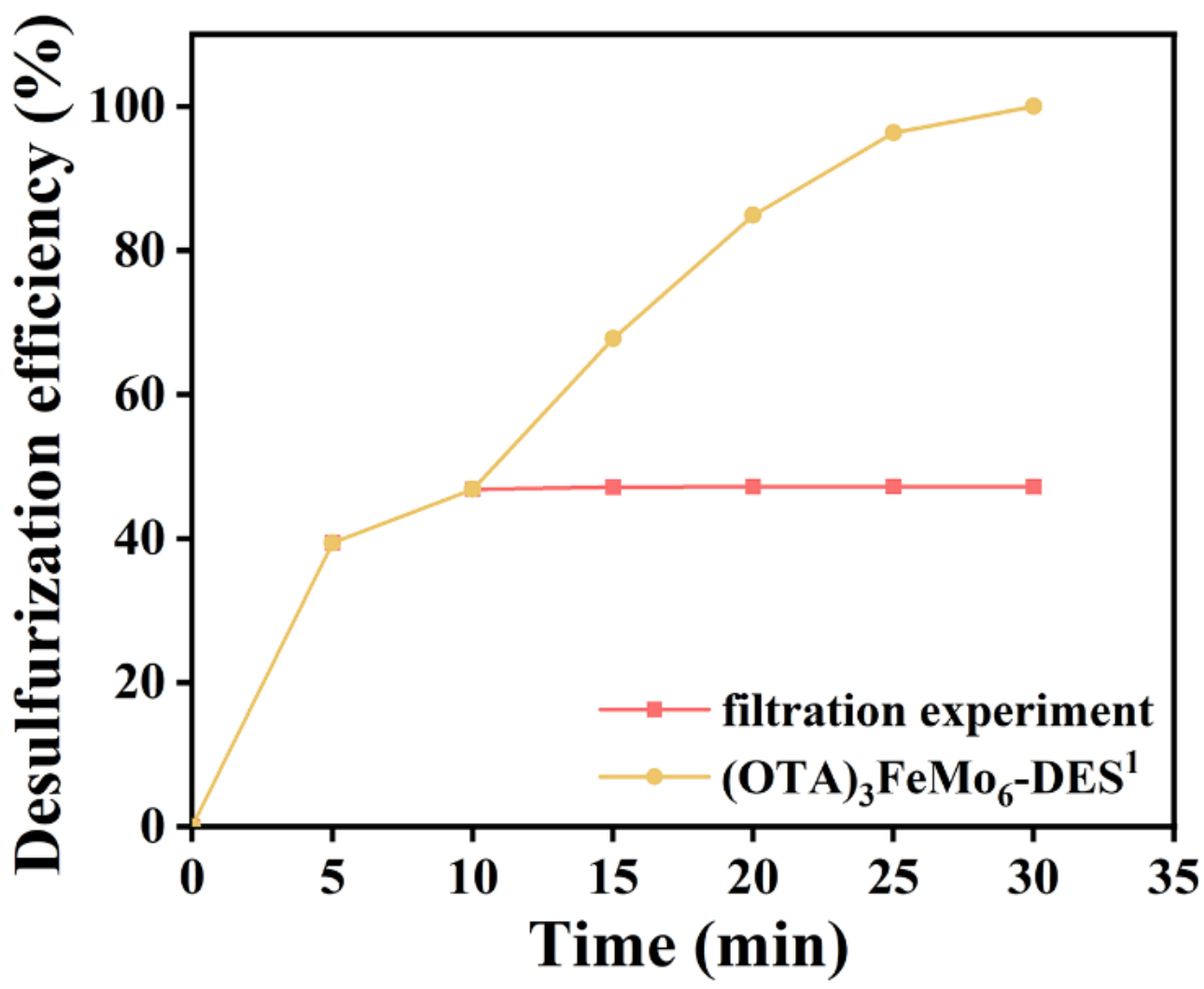

Figure. S3 DBT removal rate before and after thermal filtration.

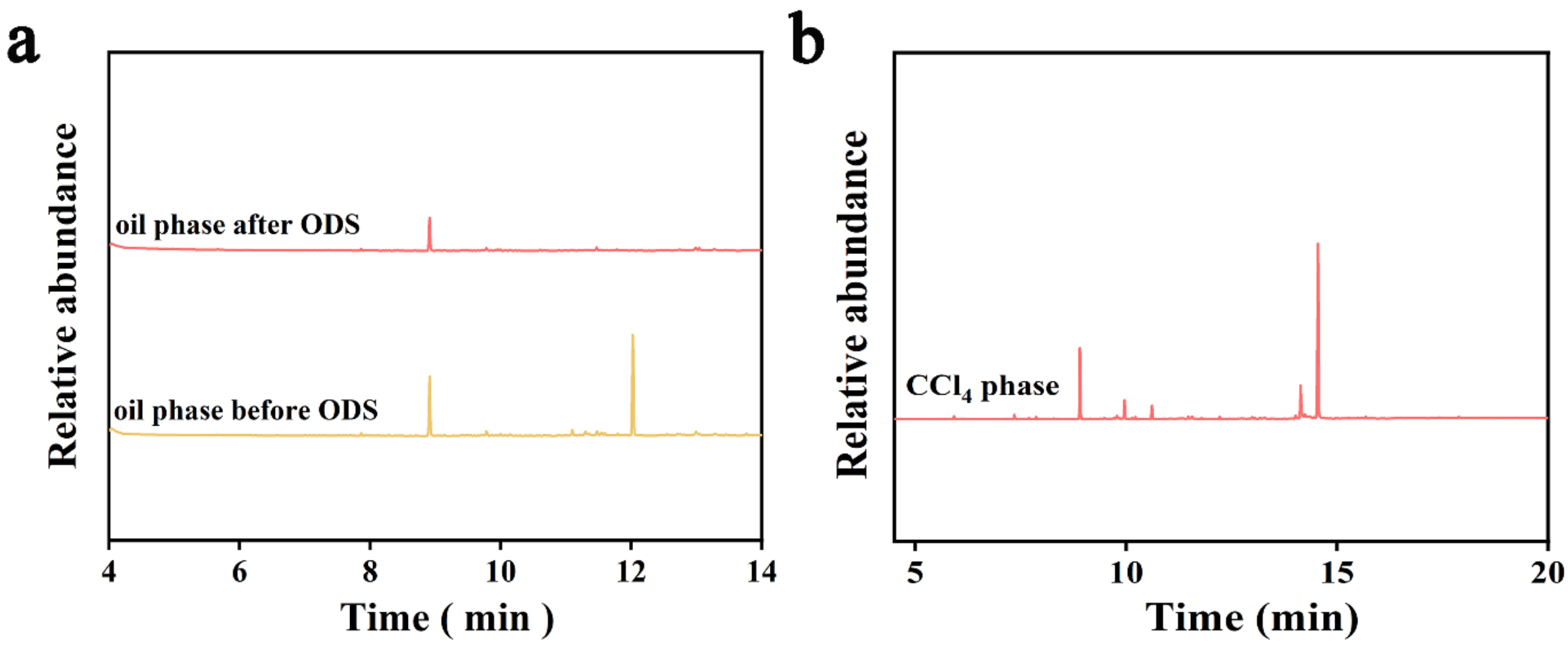

Figure. S4 GC-MS of (b) oil phase before and after reaction, (c) CCl_4 phase after mixing CCl_4 and the used $(OTA)_3FeMo_6$-DES.

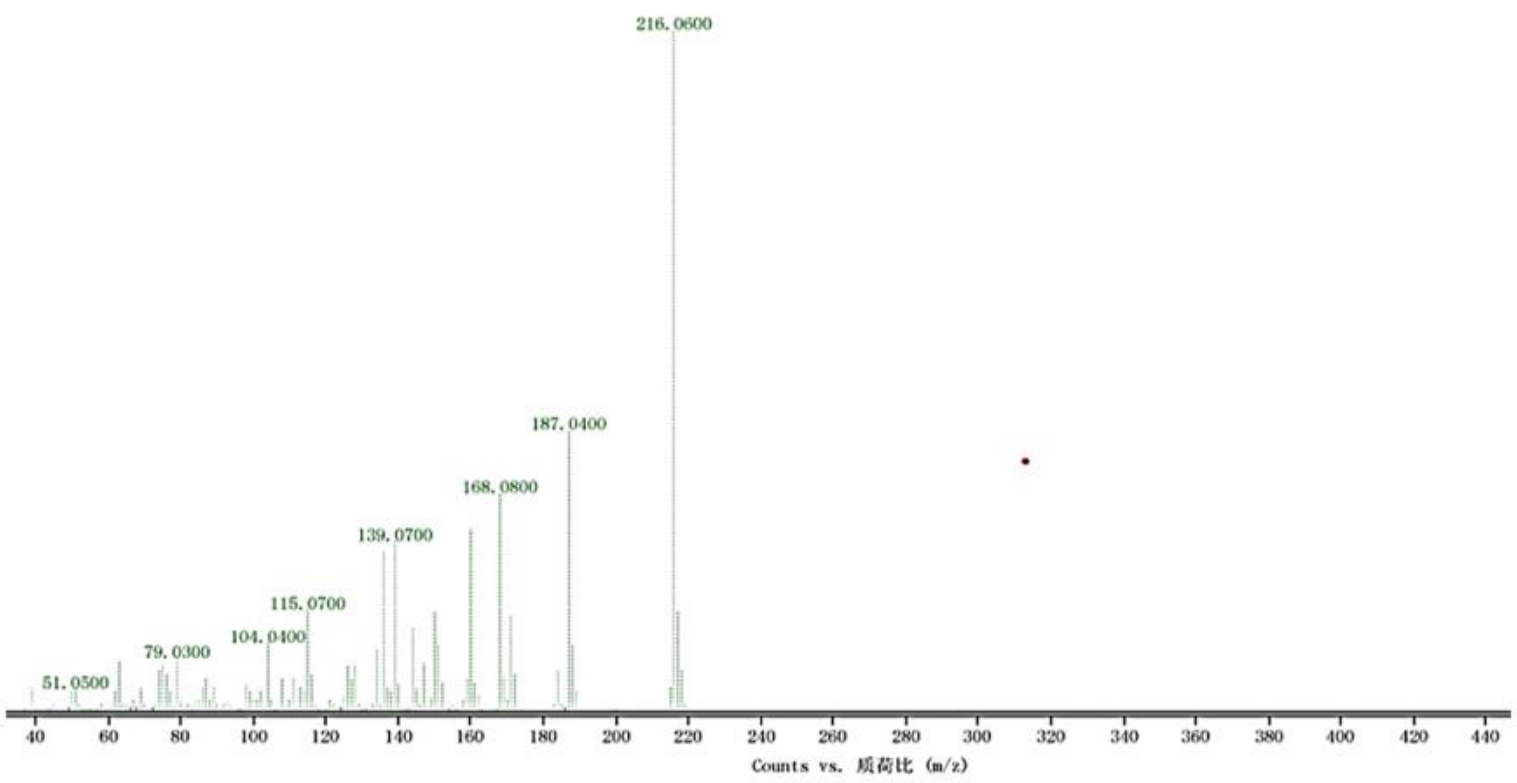

Figure. S5 Mass spectrometry corresponding to the CCl_4 phase substance.

References

[1] A. Zarnegaryan, Facile synthesis of polyoxometalate supported on magnetic g raphene oxide as a hybrid catalyst for efficient oxidation of aldehydes, Scientific Reports, 12 (2022).http://dx.doi.org/10.1038/s41598-022-21991-x

[2] N.X. Wei, W.B. Xu, S.J. Li, J.Y. Shi, Sustainable depolymerization of lignin into aromatic compounds using amphiphilic Anderson-type polyoxometalate cataly sts, International Journal of Biological Macromolrcules, 277 (2024).http://dx.doi.or g/10.1016/j.ijbiomac.2024.133257
